# Diastolic vortex ring formation in the human left ventricle: quantitative analysis using Lagrangian coherent structures and 4D cardiovascular magnetic resonance velocity mapping

**DOI:** 10.1186/1532-429X-14-S1-W30

**Published:** 2012-02-01

**Authors:** Johannes Toger, Mikael Kanski, Marcus Carlsson, Sándor J Kovács, Gustaf Söderlind, Hakan Arheden, Einar Heiberg

**Affiliations:** 1 Clinical Physiology, Skåne University Hospital, Lund University, Lund, Sweden; 2 Numerical Analysis, Centre For Mathematical Sciences, Lund University, Lund, Sweden; 3 Cardiovascular Biophysics Laboratory, Washington University Medical Center, St. Louis, MO, USA

## Summary

We show that 4D magnetic resonance velocity mapping and Lagrangian Coherent Structures can be used to quantify vortex ring volume in the human left ventricle.

## Background

The vortex ring formed in the left ventricle (LV) of the human heart during early diastolic inflow (corresponding to the Doppler E-wave) contains information about the normal and pathophysiologic aspects of diastole. Previous studies suggest that the volume of the vortex ring is an important characteristic of vortex ring formation. However, due to lack of quantitative methods, vortex ring volume has not previously been studied in the human left ventricle. The study of Lagrangian Coherent Structures (LCS) is a new flow analysis method, which for the first time enables a description of vortex ring shape and the quantification of vortex ring volume. LCS have not previously been used to quantify diastolic vortex volume in humans. Therefore, the purpose of this study was to investigate if LCS and three-dimensional, time-resolved, three-directional phase contrast magnetic resonance velocity mapping (4D PC-MR) can be used to describe vortex ring shape and quantify vortex ring volume in the human LV.

## Methods

4D PC-MR was used to measure 4D velocities over the whole heart and throughout the cardiac cycle at rest in nine healthy volunteers and one patient with dilated ischemic cardiomyopathy. From these data, LV LCS were computed in all subjects. Vortex ring volume, defined as the volume inside the LCS in the LV, was manually delineated by two independent observers at the conclusion of early rapid filling (corresponding to the end of the Doppler E-wave). Vortex ring volume was expressed as a percentage of LV volume (LVV) at diastasis.

## Results

Vortex ring volume could be delineated (see Figure) in all subjects. Vortex volume in the healthy volunteers was 51 ± 6 % of the LVV after the early filling phase, i.e. at diastasis. Interobserver variability was -1 ± 13%. In the patient, the vortex volume was 23 % of LVV.

**Figure 1:**
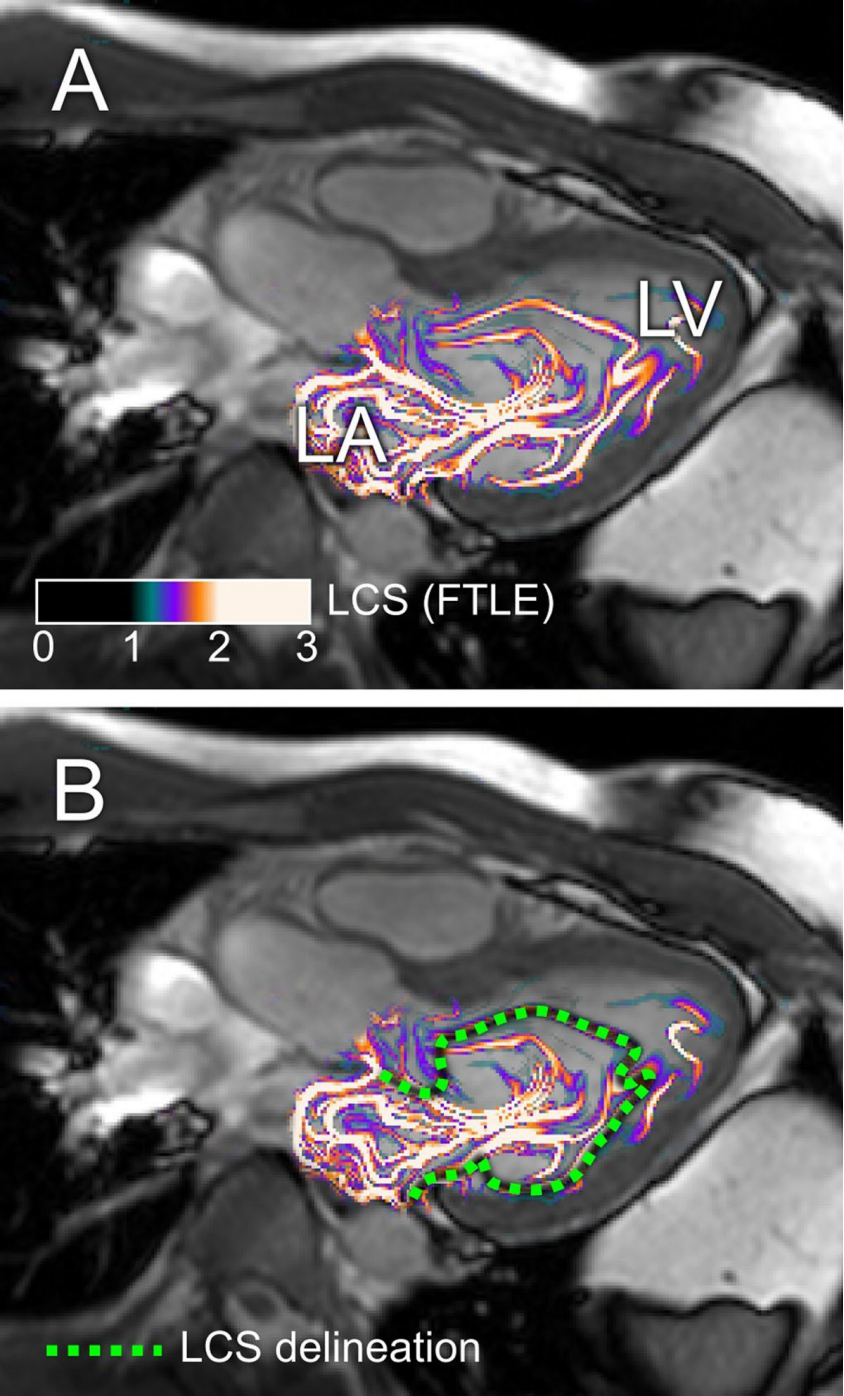
Vortex ring formation in the left ventricle after early diastolic rapid filling, as shown by Lagrangian Coherent Structures computed from 4D phase contrast velocity mapping. LCS are shown as yellow/white lines. Panel A shows a cross-section of the three-dimensional vortex ring LCS superimposed on a three-chamber view cine image of the heart, just after early diastolic rapid filling. Panel B shows the same LCS image, with a manual delineation of the vortex ring LCS. Note how the vortex ring occupies a large part of the left ventricle.

## Conclusions

Lagrangian Coherent Structures extracted from 4D cardiovascular magnetic resonance velocity mapping enables measurement of the volume of the vortex ring that develops during early rapid filling (corresponding to Doppler E-wave) of the LV. Quantitative analysis of vortex rings using LCS provides a new window into the causal physiologic relationship between diastolic function and blood flow.

## Funding

This study was supported by Swedish Research Council grants VR 621-2005-3129, VR 621-2008-2949 and VR K2009-65X-14599-07-3, National Visualization Program and Knowledge Foundation grant 2009-0080, the Medical Faculty at Lund University, Sweden, the Region of Scania, Sweden and the Swedish Heart-Lung Foundation.

SJK is supported in part by the Alan A. and Edith L. Wolff Charitable Trust, St. Louis, MO, USA, and the Barnes-Jewish Hospital Foundation, St Louis, MO, USA.

